# A Review of the Chemical Extraction of Chitosan from Shrimp Wastes and Prediction of Factors Affecting Chitosan Yield by Using an Artificial Neural Network

**DOI:** 10.3390/md20110675

**Published:** 2022-10-28

**Authors:** Ahmed Hosney, Sana Ullah, Karolina Barčauskaitė

**Affiliations:** Lithuanian Research Centre for Agriculture and Forestry, Instituto Av. 1, Akademija, 58344 Kedainiai, Lithuania

**Keywords:** shrimp shells, chitosan, chemical extraction, neural networks

## Abstract

There are two viable options to produce shrimp shells as by-product waste, either within the shrimp production phases or when the shrimp are peeled before cooking by the end user. This waste is considered a double-edged sword, as it is possible to be either a source of environmental pollution, through dumping and burning, or a promising source from which to produce chitosan as a biodegradable, biocompatible biopolymer which has a variety of agricultural, industrial, and biomedical applications. Chitosan is a deacetylated form of chitin that can be chemically recovered from shrimp shells through the three sequential stages of demineralization, deproteinization, and deacetylation. The main aim of this review paper is to summarize the recent literature on the chemical extraction of chitosan from shrimp shells and to represent the physicochemical properties of chitosan extracted from shrimp shells in different articles, such as chitosan yield, moisture content, solubility, ash content, and degree of deacetylation. Another aim is to analyze the influence of the main predictors of the chemical extraction stages (demineralization, deproteinization, and deacetylation) on the chitosan yield percentage by using a multilayer perceptron artificial neural network. This study showed that the deacetylation alkali concentration is the most crucial parameter, followed by the concentrations of acid and alkali of demineralization and deproteinization, respectively. The current review was conducted to be used in prospective studies for optimizing the chemical extraction of chitosan from shrimp wastes.

## 1. Introduction

### 1.1. Sustainable Waste Management of Shrimp Shells

During the past decades, the sustainable shrimp industry has acquired a significant importance globally and shown discernible progress, as well as an increased aquaculture production [1]. The global interest in sustainable shrimp farming has increased due to an elevating demand in the global shrimp markets and its reasonable market price, as it plays a critical economic role in supporting the food security strategy by providing a high-value protein [2]. The growth of the green economy and sustainable production are the main elements navigating the development of organic shrimp farming activities [3]. Numerous shrimp-producing countries sincerely attempt to comply with the vision of reliable aquaculture due to prompt expansion and a growing awareness of the negative impacts of shrimp farming techniques on the environment and its production [4]. Sustainable development and the implementation to improve biosecurity, increase cost efficiency, and reduce environmental degradation are the main drivers of good aquaculture practices (GAP) [5]. A substantial portion of the overall fisheries and aquaculture harvest is wasted globally, totaling approximately 35 percent of the total production [6]. In shrimp aquaculture, the shrimp shells represent a sizeable portion of the total waste, during either the molting process in shrimp production stages or during the processing of harvested shrimps when the shells and heads are removed by either the shrimp producers or by the end user before cooking [7]. Large quantities of shell wastage generated by the shrimp aquaculture industry is considered one of the most important environmental challenges due to their accumulation in the environment and their slow rate of degradation in the long term, which can lead to significant environmental impacts [8] and increase the ecological footprint produced by the shrimp aquaculture industry [9]. Dumping and burning shrimp shells are the most common non-eco-friendly methods of disposal [10].

Shrimp shells have a low economic value through their use as animal feed; however, they are rich in protein, minerals, and chitin [11].This waste could be alternatively used to produce chitin and its derivative, chitosan, which has a high economic value with low production costs [12]. One of the most economically valuable scenarios is to extract chitosan from shrimp shells, which turns shrimp shells from useless waste into economic wealth represented in chitosan as a commercially important product with a wide range of applications [13]. 

**Figure 1:**
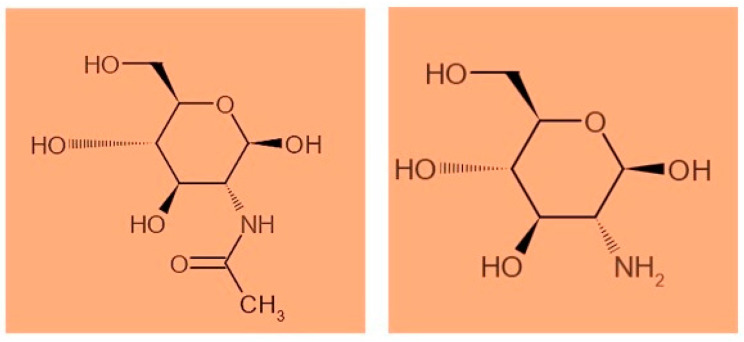
The represented structures of chitin and chitosan (de-acetylated form of chitin).

Chitosan is a natural biopolymer with biocompatibility, biodegradability, and free toxicity, which can enter different sustainable economic activities, such as food, pharmaceuticals, medicine, cosmetics, agriculture, textiles, and pulp, and the paper, biotechnology, environmental chemistry, and wastewater treatment industries to be a viable environmentally friendly option for shell remediation [14,15,16]. In recent years, scientific research on the recovery of chitosan as a valuable material from shrimp shells has been raised to mitigate the ecological footprint caused by the aquaculture industry [17]. Chitin is considered the second most abundant natural polysaccharide, after cellulose, present in the exoskeletons of crustaceans, crabs, and insects, and in fungi [18,19]. Chitosan is a chitin-partially-deacetylated derivative composed of ß-(14)-linked N-acetyl-D-glucosamine homopolymers [20], while chitin is distinguished by the existence of the acetyl group (Figure 1). Chitosan has a high molecular weight that resembles cellulose [21,22]. The main differences between chitosan and cellulose are that chitosan has an amine (-NH_2_) group in position C-2 rather than the hydroxyl (-OH) group found in cellulose; additionally, chitosan has no plant origin like cellulose [23,24,25]. 

In recent studies, chitosan is represented as a highly adaptable and promising active biopolymer [26,27]. Despite its biodegradability, it can be chemically modified to produce derivatives because it has a free amino group, which is the most useful chitin derivative with a variety of biomedical applications [27,28]. These derivatives are simple to manufacture and are commercially available [29]. Chitin and chitosan have numerous applications in the pharmaceutical industry, in tissue engineering, water treatment, agriculture, cosmetics, and in anti-tumor and anti-microbial agents [17,30]. 

### 1.2. Comparison of Chemical, Biochemical and Biological Extraction Techniques of Chitosan

Chemical, biochemical, and biological extractions can be conducted to obtain chitosan from shrimp shells [14,20,31]. Even if the chemical extraction of chitosan from shrimp shells has harmful drawbacks because of energy consumption and the production of acids and alkaline wastes, it is the most preferred commercial option [32]. The application of the chemical extraction on a large scale is due to its high efficiency in removing minerals, proteins, and acetyl groups from the shrimp waste. Moreover, the shortness of the chemical extraction time facilitates the installation of the chitosan recovery unit as an additional unit to the shrimp production lines. This develops the rapid, sustainable management of shrimp waste resulting from shrimp aquaculture, in addition to increasing the economic value of the waste to serve the circular economy principles. 

On the other hand, the biochemical and biological methods used to extract chitin and chitosan from shrimp shells are more promising green and eco-friendly techniques than chemical methods. The biochemical extraction of chitin and chitosan from shrimp shells can be conducted using organic or inorganic acids, proteolytic, and chitin deacetylase enzymes for the demineralization, deproteinization, and deacetylation stages, respectively. On the other hand, the biological recovery of chitin from shrimp shells can be performed by fermentation for the demineralization and deproteinization stages, either through lactic acid fermentation by using a selected Lactobacillus sp. strain or through non-lactic acid fermentation by using bacterial and fungal strains. The deacetylation of chitin to obtain chitosan can be conducted under the effects of the chitin deacetylase enzyme [33,34,35,36,37]. 

Even if the biochemical and biological methods are more green technologies than chemical extraction methods, there are still some obstacles to overcome for their commercial application, such as a long time consumption and high residual levels of minerals, proteins, and N-acetamido groups after the biological demineralization, deproteinization, and deacetylation processes [38,39]. The efficiency of demineralization and deproteinization could be improved by using chemical treatments or physical methods, such as sonication, heating, pulverizing, and centrifuging, to enhance the deacetylation of chitin to extract chitosan [20]. 

The urgent need that led to this review and analysis of the literature of recent studies on the chemical extraction methods and characterization of chitosan recovered from shrimp waste by different extraction methods is the lack of review articles existing on this topic. In addition, it directs the attention of scientific research toward the main affecting parameters on chitosan yield in the chemical extraction phases. The main aim of this research is to summarize the literature of recent studies on chemical extraction techniques and the characterization of chitosan that is chemically produced from shrimp shells. A multilayer perceptron artificial neural network was applied to predict the most important parameters that affect chitosan yield based on sixty-five records in reviewed articles. This is meant as a guideline to motivate researchers in the development and optimization methods of chitosan recovery from shrimp shells with high quality and economic value.

## 2. Chemical Extraction of Chitosan from Shrimp Shells

**Figure 2:**
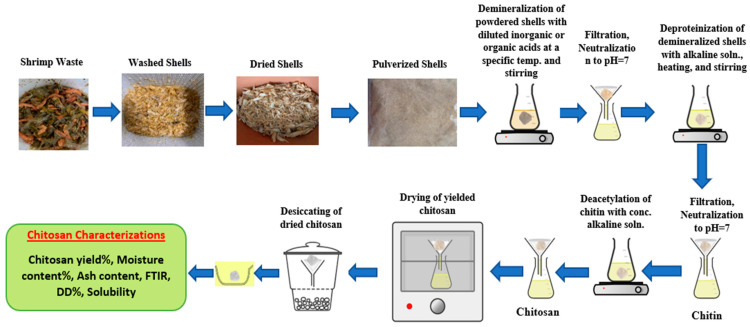
Chitin and chitosan chemical extraction from shrimp shells.

Several recent studies reported on the extraction of chitin and chitosan from raw shrimp wastes through three major processes: demineralization, deproteinization, and deacetylation. Only few researches discussed decolorization [40,41]. Figure 2 shows the chitin and chitosan extraction stages from shrimp shells. Before extracting the chitin and chitosan from shrimp wastes, the shells must be free from undesired substances, washed, dried, and pulverized, and then sieved by a 250-micron sieve before starting further extraction [40]. Chitin can be recovered from shrimp shells by removing minerals and proteins under diluted acidic and alkaline treatment, followed by a concentrated alkaline treatment to remove acetyl groups and obtain chitosan.

### 2.1. Chemical Demineralization of Shrimp Shells

Demineralization is the process of removing minerals, especially calcium carbonate. It could be conducted under the effect of inorganic or organic acids [42,43]. Therefore, demineralization is one of the most important stages in producing chitin and chitosan. It is usually obtained by inorganic acidic treatment. Shrimp shells have large amounts of minerals combined with proteins, chitin, and the rest of the exoskeleton. Calcium carbonate and calcium phosphate are the main minerals in the shells, which must be discarded to demineralize shrimp shells [31]. Most research studies reported that the demineralization process is preferred under the effect of diluted hydrochloric acid. Diluted hydrochloric acid can demineralize the shells by converting carbonate salts into chloride salts and carbon dioxide. Some studies performed the demineralization of shrimp shells by using organic acids, such as acetic and citric acids, through single or double demineralization steps [20,44,45]. The mineralization degree of shrimp shells, acid concentration, extraction temperature, and time are the main parameters affecting the efficiency of demineralization. Acid concentration is the most essential factor for controlling the removal of minerals. The pH neutralization of demineralized shells is crucial to stop the demineralization reaction. The quality of chitin production depends on the efficiency of the demineralization process, as the lower the mineral content, the higher quality of chitosan [20]. The demineralization of shrimp shells has been reported in recent literature under acidic treatment, ranging from a 1% to a 50% acid concentration for 1–24 h at 22–90 °C [8,9,10,11,12,13,14,15,16,17,18,19,20,21,22,23,24,25,26,27,28,29,30,31,32,38,39,40,41,42,43,44,45,46,47,48,49,50,51,52,53,54,55,56,57,58,59,60,61,62,63,64,65,66,67,68,69,70,71,72,73,74,75,76,77].

### 2.2. Chemical Deproteinization of Demineralized Shells

Chemical deproteinization is the process in which the chemical bonds between chitin and proteins are disrupted, and the biopolymer is depolymerized under the effects of an alkaline solution. Chemical deproteinization is an essential stage for removing the proteins from shrimp shells. Chemical deproteinization can be carried out either after or before the demineralization stage. Although deproteinization can be conducted under the effects of different alkaline solutions or chemical reagents, sodium hydroxide is the most preferred alkaline solution to use to disrupt the bonds between the chitin and proteins for protein removal or biopolymer hydrolysis. Non-optimized conditions for deproteinization could lead to a partial deacetylation of chitin. Alkali concentration, reaction temperature, and recovery time are the main drivers of the deproteinization process. The incomplete removal of protein affects the quality of chitin and chitosan and restricts their biomedical and pharmaceutical applications [20,46]. The conditions of the deproteinization of shrimp waste have been reported in recent studies under alkali concentrations ranging from 1% to 10% for 1–24 h at 22–100 °C [8,9,10,11,12,13,14,15,16,17,18,19,20,21,22,23,24,25,26,27,28,29,30,31,32,38,39,40,41,42,43,44,45,46,47,48,49,50,51,52,53,54,55,56,57,58,59,60,61,62,63,64,65,66,67,68,69,70,71,72,73,74,75,76,77].

### 2.3. Chemical Deacetylation of Chitin

Chitosan can be yielded by the chemical deacetylation of chitin, whereas the -NH_2_ group replaces the acetyl group of C2 glucosamine [47]. The degree of acetylation is the differentiation factor between chitin and chitosan. The Deacetylation of chitin can be achieved using either acidic or alkaline solutions. However, deacetylation by the acidic medium is not the preferred option because it damages the glycosidic bonds and breaks the polymer chain. On the other hand, the concentrated alkaline deacetylation of chitin is a more efficient process for removing acetyl groups. Most of the literature reported conducting the deacetylation of chitin with a concentrated sodium hydroxide solution. The quality of the chitosan is directly proportional to the degree of deacetylation, which depends on the alkali concentration, source of chitin, temperature, and time of chemical deacetylation [20]. The deacetylation conditions of chitin have been reported in recent researches using concentrated sodium hydroxide using concentrations ranging from 30% to 65% for 40 min to 72 h at 22–100 °C [8,9,10,11,12,13,14,15,16,17,18,19,20,21,22,23,24,25,26,27,28,29,30,31,32,38,39,40,41,42,43,44,45,46,47,48,49,50,51,52,53,54,55,56,57,58,59,60,61,62,63,64,65,66,67,68,69,70,71,72,73,74,75,76,77]. 

### 2.4. The Influence of Chemical Extraction Stages on Chitin and Chitosan Yield Percentage

Different authors have reported chemical demineralization, deproteinization, and deacetylation under various conditions of temperature, time, and acid and alkali concentrations. The variation of demineralization and deproteinization conditions in different articles resulted in different chitin and chitosan yields, even if some experimental studies did not report the chitin and chitosan yield percentages in their results [8,18,23,48].

Research on the black tiger shrimp (Penaeus monodon) was carried out to obtain chitin and chitosan from shrimp co-products (shells). Two different methods were applied in this study to extract chitin and chitosan from the shrimp co-products. In the first method, different treatments of 50 mL HCl were applied (3%, 4%, and 5%) at room temperature with continuous stirring at 150 rpm for 90–120 min, while deproteinization was conducted using 6% NaOH for 60 min at room temperature to produce chitin percentages 23.43 ± 0.21, 22.70 ± 0.20, and 22.13 ± 0.15. Chemical deacetylation to recover chitosan from chitin was applied by the suspension of 1 g of each chitin sample in 50 mL of 50% sodium hydroxide solution at room temperature which was stirred at 150 rpm for 90 min. The solids were filtered off using a 250-micron sieve with deionized water until neutralization, then dried at 70 °C to obtain 21.44 ± 0.19, 20.75 ± 0.12, and 20.07 ± 0.15 percentages of chitosan yield. In the second method, the same concentrations of 50 mL HCl were used (3%, 4%, and 5%) under heating of 60 °C with continuous stirring at 150 rpm in a water bath for 90–120 min., while the deproteinization was performed under 5% NaOH for 60 min in a water bath under heating of 60 °C with constant stirring at 150 rpm to obtain 25.70 ± 0.30, 25.33 ± 0.20, and 24.37 ± 0.30 percentages of chitin. Then, the chitin was deacetylated under the same conditions as the first method, except the heating was at 60 °C to obtain chitosan yield percentages of 23.24 ± 0.24, 22.97 ± 0.15, and 22.40 ± 0.26 [17].

A research study has applied chemical extraction using three levels of diluted HCl (2%, 3%, and 4%) at ambient temperature (28 ± 2 °C) to demineralize the shrimp waste for 16 h, while chemical deproteinization was conducted under a unique concentration of 4% sodium hydroxide for 20 h to produce three different yields of chitin at 17.36%, 14.02%, and 13.12%. The deacetylation of chitin was applied by using four different concentrations (30%, 40%, 50%, and 60%) of NaOH at 65 °C for 20 h to obtain a 15.4% of chitosan yield [49].

In contrast, another researcher performed the chemical deproteinization before the demineralization as an initial phase. The deproteinization was conducted with 3.5% NaOH at 90 °C with continuous stirring for 1 h, followed by chemical demineralization under the effect of 6% HCl at 90 °C for 1 h; the obtained chitin yield was 40%. Then, chitin was converted to chitosan under the effect of 50% NaOH at various heating temperatures and times (70, 80, 90, and 100 °C for 40, 60, and 80 min, respectively) to obtain higher yield percentages of chitosan at 83.74, 72.17, 61, and 54.66 [50]. 

An optimization study applied the chemical method to optimize the recovery of chitin and chitosan from shrimp shells at ambient temperature under different concentrations of acids and bases for demineralization and deproteinization, respectively. Five treatments of hydrochloric acid (10%, 20%, 30%, 40%, and 50%) were applied to chemically demineralize the shrimp shells to produce five samples of demineralized shrimp shells. For each demineralized sample, the chemical deproteinization was conducted using four levels of sodium hydroxide (1.5%, 3%, 6%, and 8%) to obtain twenty yields of extracted chitin with different yield percentages. Then, the chitin yields were treated overnight with 50% sodium hydroxide at 60–70 °C heating temperature for the chitin deacetylation to produce the various ranges of chitosan yield percentages (Table 1). The optimization process found that the optimal concentration of hydrochloric acid and sodium hydroxide to produce good quality white chitin and chitosan yield with lower acidic and alkaline residual impact on the environment was 30% and 6%, respectively [51].

Another technique of the chemical extraction of chitin and chitosan was conducted by boiling the collected shrimps in salty water for 10 min, peeling them with an automated machine and separating the shells from the meat, drying them at 50 °C in a dry oven for 24 h, grinding them in a laboratory mixer, and storing them at −25 °C for further recovery processing. The first phase of the recovery process of chitin and chitosan from shrimp shells was the deproteinization, conducted by adding 30 mL of 2% NaOH solution to 1 g of shells at 90 °C for 2 h, and then centrifuging them at 4000 rpm for 15 min to separate the alkali-insoluble fraction. Then, a reflux of 10% v/v acetic was applied at 60 °C for 6 h. Then, 10.8% chitin was separated under the effect of centrifugal power at 4000 rpm for 15 min, and 8.2% of chitosan was yielded under the effect of 8% NaOH solution at pH = 9 [52]. 

Other researchers have prepared shrimp shells in three different particle sizes, 16, 32, and 60 mesh knit, to obtain chitin and chitosan. Chemical demineralization was conducted using 2% HCl at 30 °C for 12 h. This was followed by separating the alkali-insoluble fraction by centrifugation at 4000 g for 15 min. Then, the precipitate was neutralized with distilled water. Chemical deproteinization was applied under the effect of 4% NaOH alkaline solution at 90 °C for 12 h to obtain three diverse levels of chitin: 79.4%, 74%, and 42.2% for 16, 32, and 60, respectively. While the deacetylation of the three different chitin samples has been conducted with a new technique under the effect of 45% NaOH in a microwave oven for irradiation at 600 watts using two methods. The first method was continuous irradiation for 15 min to obtain 44.8%, 52.2%, and 44.4% yields of chitosan. The second method applied pulsed irradiation six times, with stirring after 5 min of each irradiation pulse to produce 43%, 32.2%, and 42.4% yields of chitosan [53]. 

The sequence of demineralization and deproteinization has a significant impact on chitin and chitosan yield. reported that the sequence of demineralization and deproteinization stages has a direct impact on chitin and chitosan yield, whereas the beginning chemical extraction with demineralization followed by deproteinization is highly recommended [46,54].

### 2.5. Characteristics of Chitosan Chemically Obtained from Shrimp Shells

Recent articles have discussed the synthesis of chitosan from shrimp shells and showed that chitosan has a variety of significant physicochemical properties and biological functionality [55], such as biodegradability, bioactivity, non-toxicity, active linear amino polysaccharide with a high nitrogen content with complexing and chelating ability, hydrophilicity, hypolipidemic, water insolubility, solubility in diluted organic and inorganic acids, crystallinity, cross-linking and chemical activation through its reactive groups, ionic conductivity, polyelectrolyte properties in an acidic medium, salt formation with organic and inorganic acids, positively charged biopolymers acting as a flocculating agent regarding its ability to interact with negatively charged molecules, absorbency, adhesivity, biocompatibility, blood anticoagulant properties, antitumor and antimicrobial activities, film-forming ability, and high viscosity regarding its ability to form intermolecular hydrogen bonds [56,57]. 

The influence of the DD (degree of deacetylation) and solubility degree are the main crucial factors in determining the quality of the physicochemical and biological characteristics of chitosan, depending on the nature, species, and the techniques used in the chitosan synthesis from chitin and the optimization of the extraction processes (demineralization, deproteinization, decolorization, and deacetylation) and their conditions (concentrations, temperature, and time). The DD (degree of deacetylation) is represented by the number of free amino groups (-NH_2_) in chitosan as polysaccharides and can be used to indicate the difference between chitin and chitosan through the number of acetyl groups removed from chitin to form chitosan. The removal of acetyl groups affects the physicochemical properties of chitin and chitosan and their appropriate applications in different fields [48,58,59]. The testing of chitosan characteristics, according to standard methods, is very important in order to confirm chitosan synthesis, purity, and yield percentage. In most of the previous studies, chitosan physicochemical properties were analyzed by using various analytical techniques to ensure the quality of chitosan by the determination of the following parameters [60].

#### 2.5.1. Moisture Content Determination

Moisture content, after producing chitosan from shrimp shells, can be determined using the gravimetric method [61]. The moisture content percentage may be varied, depending on indoor or outdoor (sunlight) aquaculture, climatic season, and relative humidity. Moisture content is recommended to be less than 10 for commercial chitosan [62]. The variation of the moisture content, heterogeneity, and storage period of shrimp waste samples might lead to discrepancies in the characteristics and quality of chitosan. The existence of the water phase has a significant impact on the flowability, shelf life, and compressibility of chitosan solid-based formulations [63]. In recent studies, different researchers reported varied moisture content percentages ranging from 1.25–8.71 (Table 2). The moisture content of chitosan extracted from shrimp shells can be calculated according to the following formula [64]:

(1) $$\mathrm{Moisture}\;\mathrm{content}\;\%=\frac{\mathrm{wet}\;\mathrm{Weight}\;–\;\mathrm{dry}\;\mathrm{Weight}}{\mathrm{wet}\;\mathrm{Weight}}\times 100$$

#### 2.5.2. Solubility of Chitosan

**Figure 3:**
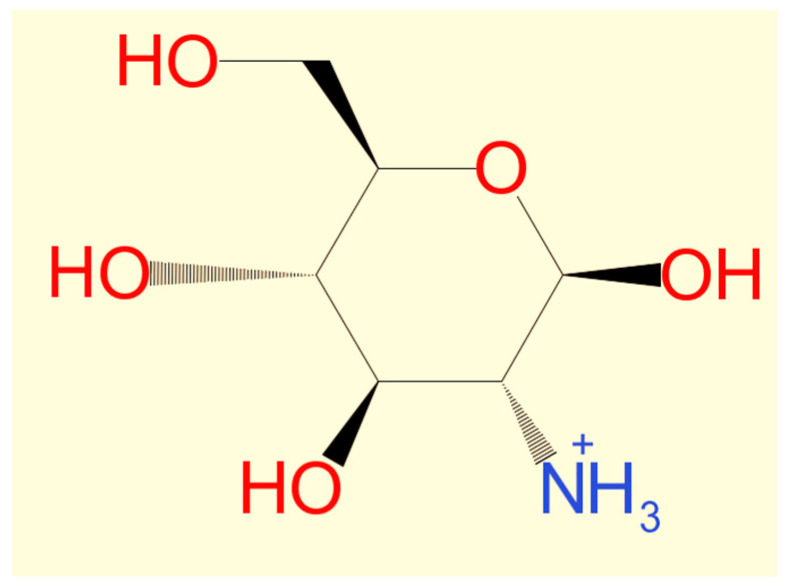
Cationic soluble form of chitosan.

**Figure 4:**
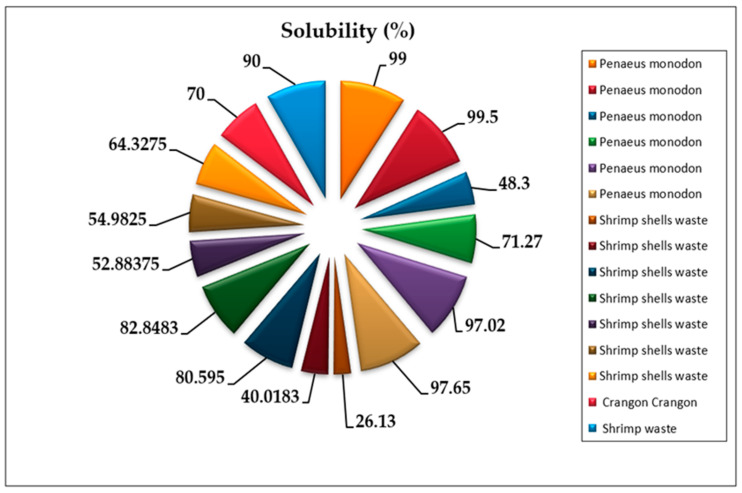
Solubility of chitosan recovered from shrimp shells in 15 experiments from reviewed articles [17,45,49,50,68].

Chitosan’s solubility is considered one of the most crucial parameters for determining the quality of chitosan obtained from shrimp shells, where the higher solubility indicates a higher purity and quality of chitosan [65]. The solubility of chitosan could be affected by the conditions of the deacetylation reaction of chitin (temperature, time, and alkali concentration) [66]. For example, when the deacetylation of chitin from the same shrimp species was conducted at the same concentration of concentrated NaOH under various heating duration and temperatures, the solubility of the chitosan was different [17,50]. In contrast, other studies reported that the solubility of chitosan varied under different alkali concentrations at stable heating conditions [49]. Moreover, solubility might be affected by the chemical concentrations and conditions of the pre-treatment of shells to obtain chitin (demineralization and deproteinization) [67]. The recent literature showed that the solubility of chitosan depends on the degree of deacetylation. Chitosan is a weak base insoluble in water and organic solvents, even if its solubility can be shown by dissolving chitosan powdered extract in diluted acetic acid [68]. Diluted aqueous acetic acid converts insoluble glucosamine units of chitosan into a cationic soluble form [69] (Figure 3). Chitosan’s solubility in reviewed manuscripts ranges from 26.13% to 99.5% [17,45,49,50,68] (Figure 4).

#### 2.5.3. Ash Content Percentage

**Figure 5:**
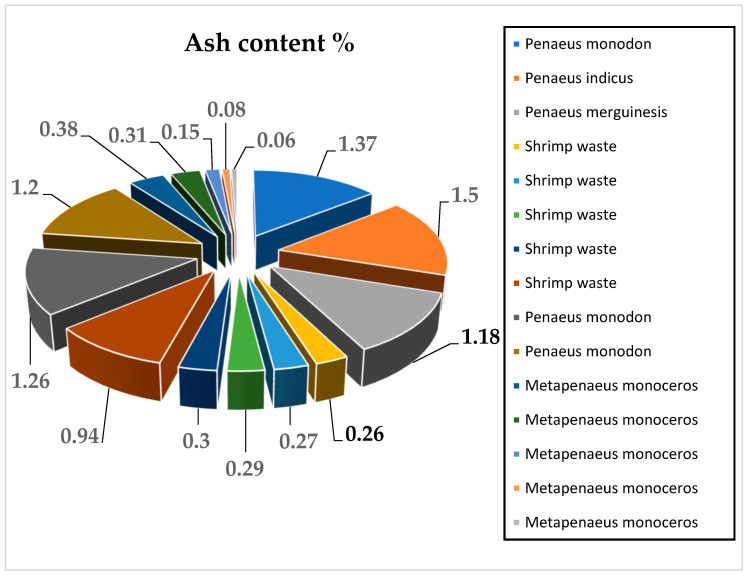
Ash content (%) of chitosan extracted from shrimp shells by the chemical method in 15 experiments from reviewed articles [8,17,48,49,50,51].

The total ash content percentage of chitosan extracted from shrimp shells equals the amount of ash in chitosan divided by the weight of the chitosan sample multiplied by 100%. The ash content percentage of chitosan is an important indicator of the performance and effectiveness of the demineralization process for mineral and carbonate removal and should not exceed 1% for high-quality chitosan. The reduction of the ash content of shrimp shells by demineralization can be used as a quality estimator for chitosan purity. The higher the reduction of the ash content, the higher the purity degree of the chitosan [70]. The reported results of different research studies showed that the ash content of chitosan extracted from shrimp shells ranged between 0.27% and 1.5%, as represented in Figure 5 [8,17,48,49,50,51].

#### 2.5.4. FT-IR (Fourier Transform Infrared)

FTIR (Fourier transform infrared spectroscopy) is based on the fact that almost all of the molecules can absorb infrared light in the region of the electromagnetic spectrum, and that gives FTIR the ability, as a sensitive advanced analytical technique, to identify and characterize most organic substances through the identification of the functional groups, side chains, and cross links in the organic molecular groups and compounds in the spectral ranges of 4000–400 cm^−1^. 

In recent studies, FTIR was used to determine the degree of deacetylation of chitosan extracted from shrimp waste. The primary bonds of standard chitosan in the FT-IR spectra are 3858 cm^−1^ for OH stretching, NH stretching 3609 cm^−1^, CH stretching 2862 cm^−1^, amide I band 1643 cm^−1^, amide II band 1552 cm^−1^, CH_2_ bending 1421 cm^−1^, CO- stretching 1022 cm^−1^, CH_3_ wagging alone chain 752 cm^−1^, and NH-out of plane bending 564 cm^−1^ [71]. The recent literature confirmed the chitosan formation by FT-IR spectra vibrational modes, as represented in Table 3. 

#### 2.5.5. Degree of Deacetylation (DD%)

The degree of acetylation is one of the most important parameters that determine the quality of chitosan. The higher the purity of the chitosan, the higher the degree of deacetylation. The deacetylation degree is always reported as an important factor for determining the biological activity, polymeric and physicochemical characteristics, and biomedical applications of chitosan [72,73,74,75]. Moreover, the degree of deacetylation is a crucial indicator of the effectiveness of removing acetyl groups by chemical deacetylation. Even if the degree of the deacetylation of commercial chitosan ranges from 70% to 85% [76], the recent studies of chitin’s deacetylation to chitosan, reviewed in this research, showed that DD% ranged between 39.1% and 97% (Table 4), depending on all the conditions used in chitosan production from shrimp waste. It was reported that the sodium hydroxide concentration is the main driver of the deacetylation stage, with a minimum concentration of 40%, whereas the degree of deacetylation increases with the increase of alkali concentration. In addition, the increase in reaction time leads to an increase in the deacetylation of chitin to obtain chitosan; however, it reduces the intrinsic viscosity and molecular weight of the yielded chitosan [77].

## 3. Neural Network Modeling (Multilayer Perceptron) of Chitosan Yield

**Figure 6:**
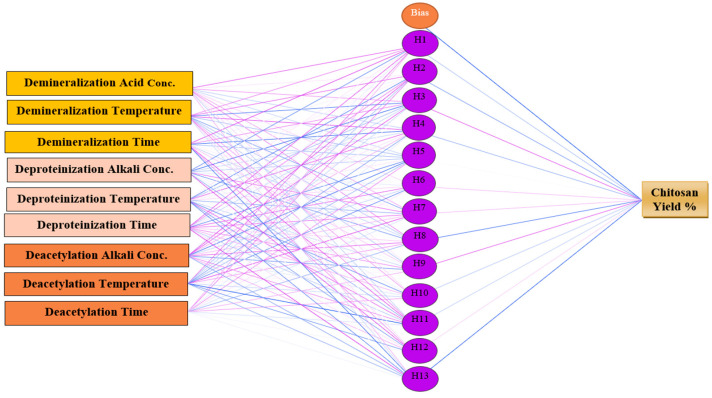
Multilayer perceptron neural network architectural graph of chitosan yield (%).

The multilayer perceptron is one of the most reported artificial neural network architectures in the literature, which can be used in chemical research to explore the complexity of nonlinear relationships between chemical, physical, or biological parameters [78,79,80,81]. Neural network architectures have many advantages over conventional methods and semi-empirical models, which require large numbers of input parameters. In contrast, neural network architectures use a minimal number of measured parameters to predict changes in the required parameters with high accuracy levels [82,83,84,85,86]. The multilayer perceptron artificial neural network consists of input signals which contain the independent variables, an output neural layer containing the dependent variable, and one or more hidden layers. The data are transmitted from the input layer directly to the output layer, such as the human brain, which transfers signals in a single direction. Hidden layers create indirect relationships between input and output variables due to large numbers of hidden nodes [87,88,89,90,91]. In the present study, a multilayer perceptron artificial neural network was fitted to the data reported in 65 records in reviewed articles (Figure 6) on demineralization, deproteinization, and deacetylation parameters as input independent variables and chitosan yield percentage as a dependent variable to predict the main influencing parameters on chitosan yield by using IBM SPSS Statistics for Windows, Version 25.0. (Armonk, NY, USA)

## 4. Results of Neural Network Modeling (Multilayer Perceptron) of Chitosan Yield

Different models were tested before the final multilayer perceptron artificial neural network architecture was confirmed, resulting in altered numbers of hidden nodes. Ultimately, a multilayer perceptron artificial neural network architecture model was applied with a total of nine input nodes for the chemical extraction stages and thirteen hidden nodes in terms of predictions of chitosan yield. The hyperbolic tangent and SoftMax functions were applied for the hidden layer and the output layer, respectively, and the error function was expressed by cross entropy (Table 5). The model summary (Table 6) shows the training and testing samples. The procedure was carried out until one consecutive step was obtained with no decrease in the error function of the test sample. Moreover, it shows the values of the cross-entropy error are 0.54 and 0.975 for the training and testing sample, respectively, while the percentage of incorrect predictions in the training and testing sample are 1.7% and 3.1%, respectively. The small value of cross entropy can indicate the model fitting power, as the smaller the cross-entropy error, the more the powerful model is.

**Figure 7:**
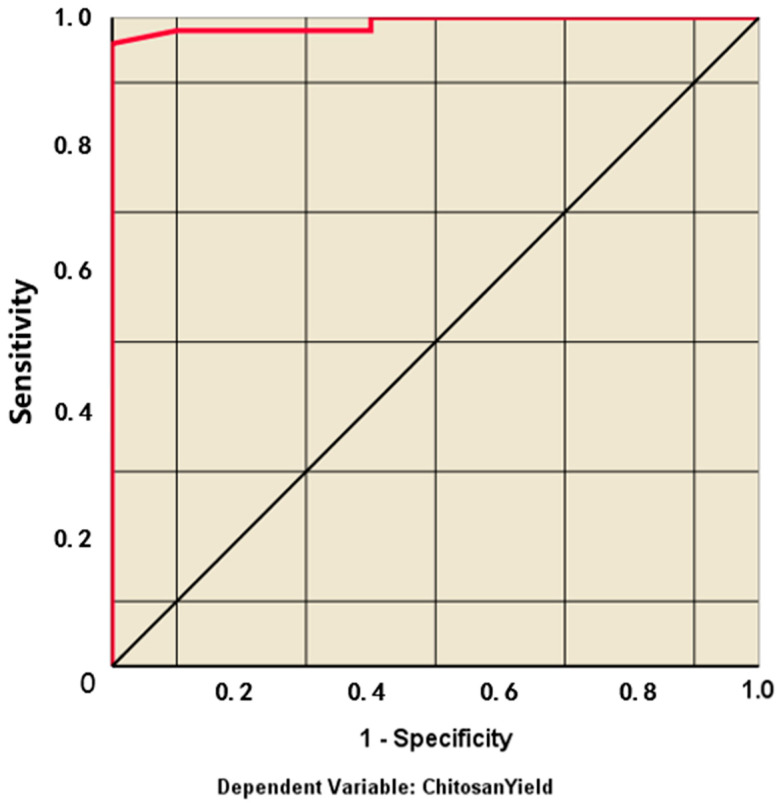
ROC curve of the multilayer perceptron artificial neural networks.

**Figure 8:**
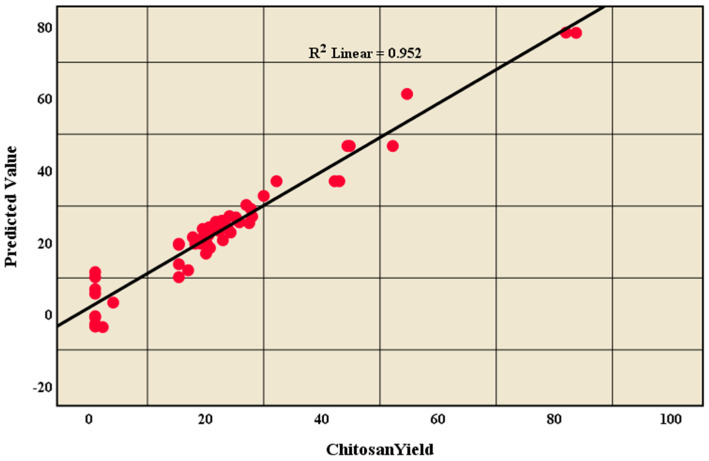
Linear relationship between chitosan yield in reviewed articles with the predicted value.

The sensitivity (true positive rate) versus specificity (false positive rate) at all possible cut-offs of the parameter can be represented by the ROC curve. Each combination of the sensitivity and specificity as a pair corresponds to a given result or decision threshold. As shown in Figure 7, the ROC curve is based on the pair of training and testing samples closer to the upper-left corner, and the area under the curve (AUC) is 0.989, which indicates the high accuracy of the test. Moreover, Figure 8 represents the linear relationship between the chitosan yield in reviewed articles and the predictive value (R^2^=0.952) to confirm that the data has a powerful fitting to the model.

**Figure 9:**
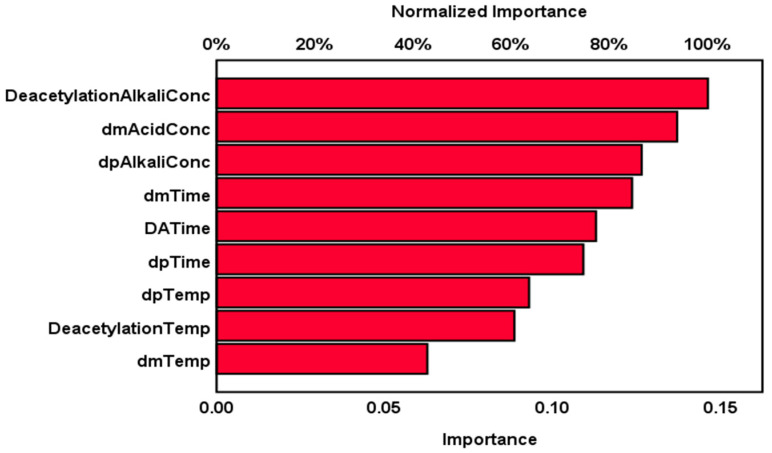
Independent variables important chart of chemical extraction parameters.

The impact of the independent parameters of demineralization, deproteinization, and deacetylation on chitosan yield in the multilayer perceptron artificial neural network architecture model is shown in Figure 9, which indicates the relative and normalized importance of each independent variable. The chart shows that the top three influencing factors in the chemical extraction process on chitosan yield are deacetylation alkali concentrations, demineralization acid concentration, and deproteinization alkali concentration (with normalized importance of 100.0%, 93.7%, and 86.5%, respectively), while the time of each extraction step is in the second order of importance with 84.6%, 77.2%, and 74.7% for demineralization time, deacetylation time, and deproteinization time, respectively. The multilayer perceptron artificial neural network model agreed with the literature and confirmed that the acid and alkali concentrations are the main drivers of the chemical extraction of chitosan from shrimp shells in terms of influence on chitosan yield.

## 5. Conclusions

Shrimp shells are one of the crustacean resources considered for chitin and chitosan production. It has gained attention from scientists and aquaculture researchers to develop the best management option for this kind of waste. The aim is to produce a valuable material, which has a wide spread of applications like chitosan, and to reduce the environmental impact resulting from this large portion of the aquaculture sector. This paper reviewed recent articles on the chemical extraction of chitosan from shrimp shells and detailed information regarding the pre-treatment of shrimp wastes, extraction conditions, and the chemicals used in the extraction, and their influence in each synthesis stage (demineralization, deproteinization, and deacetylation). In addition, the paper showed the variety of the ranges of physicochemical characteristics of chitosan obtained from shrimp shells in different studies. Moreover, the paper summarized the results of physicochemical properties in tabular and graphical outputs to facilitate, for the researchers, the comparison of different chemical extraction methods, materials, concentrations, conditions, and produced characteristics. A multilayer perceptron artificial neural network was applied to predict the main drivers affecting chitosan yield, based on the data given in different studies. The prediction analysis showed that the deacetylation alkali concentration is the most crucial parameter, followed by the concentrations of acid and alkali used in demineralization and deproteinization, respectively. This review was conducted to optimize the chemical extraction of chitosan from shrimp wastes in prospective research.

## Figures and Tables

**Table 1:** Chitin and chitosan yield percentage under distinctive chemical extraction conditions [51].

Demineralization Acid Concentration (%)	Deproteinization Alkali Concentration (%)	Chitin Yield %	Deacetylation Alkali Concentration (%)	Deacetylation Temperature (°C)	Chitosan Yield %
10	1.5	30.6	50	60–70	30.00
10	3	29.1	50	60–70	28.00
10	6	28.7	50	60–70	27.00
10	8	27.7	50	60–70	25.20
20	1.5	28	50	60–70	27.80
20	3	27.7	50	60–70	27.50
20	6	26.4	50	60–70	25.80
20	8	24.9	50	60–70	24.30
30	1.5	24.4	50	60–70	24.10
30	3	24	50	60–70	23.80
30	6	22.6	50	60–70	21.76
30	8	21.8	50	60–70	20.50
40	1.5	23.8	50	60–70	22.80
40	3	22.6	50	60–70	22.20
40	6	21.1	50	60–70	20.60
40	8	20.2	50	60–70	19.50
50	1.5	22.1	50	60–70	20.90
50	3	20.7	50	60–70	19.50
50	6	19.4	50	60–70	17.80
50	8	18.2	50	60–70	15.40

**Table 2:** The moisture content of chitosan extracted from shrimp waste origin by chemical method.

Crude Shells Sample	Moisture Content (%)	Reference
*Penaeus monodon*	4.3	[8]
*Penaeus indicus*	3.9	[8]
*Penaeus merguiensis*	4.05	[8]
Shrimp shell waste	4	[18]
Shrimp shell waste	4.36	[18]
Shrimp shell waste	5.02	[18]
Shrimp shell waste	6	[18]
Shrimp shell waste	5.79	[18]
Shrimp shell waste	6.49	[18]
Shrimp shell waste	5.52	[18]
Shrimp shell waste	5.21	[18]
Shrimp waste	8.71	[48]
*Penaeus monodon*	1.28	[17]
*Penaeus monodon*	1.27	[17]
*Penaeus monodon*	1.25	[17]
*Penaeus monodon*	1.26	[17]
Shrimp shell waste	8.25	[49]
Shrimp shell waste	7.69	[49]
Shrimp shell waste	8.32	[49]
Shrimp shell waste	7.96	[49]
*Metapenaeus monoceros*	8.01	[51]
*Metapenaeus monoceros*	7.53	[51]
*Metapenaeus monoceros*	7.44	[51]
*Metapenaeus monoceros*	7.31	[51]
*Metapenaeus monoceros*	6.62	[51]

**Table 3:** Fourier transform infrared spectroscopic analysis of chitosan extracted by the chemical method in reviewed articles.

	Vibration Modes						
Reference	NH_2_, OH in Pyranose Ring (cm^−1^)	-NH Stretching (cm^−1^)	Symmetric (CH_3_) and Asymmetric (CH_2_) Stretching (cm^−1^)	Amide I Amide II ** and Amide III *** Bands (cm^−1^)	CH_2_ Bending and CH_3_ Deformation (cm^−1^)	CO Stretching (cm^−1^)	Ring Stretching (cm^−1^)
[8]	3438	---	2924	1421 ***	---	1075	896
[18]	3452.58	---	2924.01	1622.21 1550 **	1446	1078	875
[68]	3398	3262	---	1658	---	---	876
[45]	3846	3295	2878	1646 1537 **	1403	1018	---
[69]	3450.65	2852.72	2924.09	1629.85	---	---	---
[70]	3411	3425	2919	1651 1321 ***	1418	1078	899
[71]	3425	2881	2921-2879	1647.19 1559 **	---	---	---

--- No band found.

**Table 4:** Degree of deacetylation percentages (DD %) of chitosan extracted from shrimp waste by chemical methods.

Shell Origin	DD (%)	Extraction Condition Minutes	Reference
Shrimp shell waste	39.10	2% HCl for DM, 4% NaOH for DP, and 40% NaOH for DA, at DT 65 °C	[18]
Shrimp shell waste	40.00	2% HCl for DM, 4% NaOH for DP, and 60% NaOH for DA, at DT 65 °C	[18]
Shrimp shell waste	41.00	3% HCl for DM, 4% NaOH for DP, and 40% NaOH for DA, at DT 65 °C	[18]
Shrimp shell waste	42.00	3% HCl for DM, 4% NaOH for DP, and 60% NaOH for DA, at DT 65 °C	[18]
Shrimp shell waste	61.00	4% HCl for DM, 4% NaOH for DP, and 40% NaOH for DA, at DT 65 °C	[18]
Shrimp shell waste	70.00	4% HCl for DM, 4% NaOH for DP, and 60% NaOH for DA, at DT 65 °C	[18]
Shrimp shell waste	58.00	5% HCl for DM, 4% NaOH for DP, and 40% NaOH for DA, at DT 65 °C	[18]
Shrimp shell waste	67.00	5% HCl for DM, 4% NaOH for DP, and 60% NaOH for DA, at DT 65 °C	[18]
*Penaeus monodon*	45.50	(2%, 3%, 4% ) HCl for DM, 4% NaOH for DP, and 30% NaOH for DA, at DT 65 °C	[49]
*Penaeus monodon*	61.24	(2%, 3%, 4% ) HCl for DM, 4% NaOH for DP, and 40% NaOH for DA, at DT 65 °C	[49]
*Penaeus monodon*	79.57	(2%, 3%, 4% ) HCl for DM, 4% NaOH for DP, and 50% NaOH for DA, at DT 65 °C	[49]
*Penaeus monodon*	81.24	(2%, 3%, 4% ) HCl for DM, 4% NaOH for DP, and 60% NaOH for DA, at DT 65 °C	[49]
Shrimp shell waste	76.26	6% HCl for DM, 3.5% NaOH for DP, and 50% NaOH for DA, at DT 70 °C	[50]
Shrimp shell waste	81.37	6% HCl for DM, 3.5% NaOH for DP, and 50% NaOH for DA, at DT 80 °C	[50]
Shrimp shell waste	81.25	6% HCl for DM, 3.5% NaOH for DP, and 50% NaOH for DA, at DT 90 °C	[50]
Shrimp shell waste	84.87	6% HCl for DM, 3.5% NaOH for DP, and 50% NaOH for DA, at DT 100 °C	[50]
*Litopenaeus vannamei*	81.00	DM with 2% HCl for shrimp shells with 16 mesh size, then DP under 4% NaOH, and 45% NaOH for DA, at 600 watts in the microwave for 15 min	[53]
*Litopenaeus vannamei*	72.00	DM with 2% HCl for shrimp shells with 32 mesh size, then DP under 4% NaOH, and 45% NaOH for DA, at 600 watts in the microwave for 15 min	[53]
*Litopenaeus vannamei*	78.00	DM with 2% HCl for shrimp shells with 60 mesh size, then DP under 4% NaOH, and 45% NaOH for DA, at 600 watts in the microwave for 15 min	[53]
*Litopenaeus vannamei*	81.00	DM with 2% HCl for shrimp shells with 16 mesh size, then DP with 4% NaOH, and 45% NaOH for DA at 600 watts in the microwave for six pulses of 5 min	[53]
*Litopenaeus vannamei*	92.00	DM with 2% HCl for shrimp shells with 32 mesh size, then DP with 4% NaOH, and 45% NaOH for DA at 600 watts in the microwave for six pulses of 5 min	[53]
*Litopenaeus vannamei*	89.00	DM with 2% HCl for shrimp shells with 60 mesh size, then DP with 4% NaOH, and 45% NaOH for DA at 600 watts in the microwave for six pulses of 5 min	[53]
Pink shrimp shells	97.00	3% HCl for DM, 4% NaOH for DP, heating at 2-atmospheric pressure in the autoclave for 1 h and then steeping in 40% NaOH for DA for 4 days	[77]
Brown shrimp shells	92.00	3% HCl for DM, 4% NaOH for DP, heating at 2-atmospheric pressure in the autoclave for 1 h and then steeping in 40% NaOH for DA for 4 days	[77]
Pink shrimp shells	94.00	3% HCl for DM, 4% NaOH for DP, and 40% NaOH for DA, and then autoclaved at 2-atmospheric pressure for 3 h	[77]
Brown shrimp shells	90.00	3% HCl for DM, 4% NaOH for DP, and 40% NaOH for DA, and then autoclaved at 2-atmospheric pressure for 3 h	[77]

Where DM, DP, DA, and DT are demineralization, deproteinization, deacetylation, and deacetylation temperature, respectively.

**Table 5:** Network information of the multilayer perceptron artificial neural networks.

Network Information			
Input Layer	Factors	1	Demineralization acid concentration
		2	Demineralization temperature
		3	Demineralization time
		4	Deproteinization acid concentration
		5	Deproteinization temperature
		6	Deproteinization time
		7	Deacetylation alkali concentration
		8	Deacetylation temperature
		9	Deacetylation time
	Number of Units		84
Hidden Layer(s)	Number of Hidden Layers		1
	Number of Units in Hidden Layer		13
	Activation Function		Hyperbolic tangent
Output Layer	Dependent Variables	1	Chitosan yield
	Number of Units		2
	Activation Function		SoftMax
	Error Function		Cross-entropy

**Table 6:** Summary of the multilayer perceptron artificial neural network model.

Model Summary		
Training	Cross Entropy Error	0.54
	Percent Incorrect Predictions	1.7%
	Stopping Rule Used	1 consecutive step with no decrease in errors
	Training Time	0:00:00.05
Testing	Cross Entropy Error	0.975
	Percent Incorrect Predictions	3.1%

## Data Availability

No new data were created or analyzed in this study. Data sharing is not applicable to this article.
